# In Silico Optimization of Fiber-Shaped Aerosols in Inhalation Therapy for Augmented Targeting and Deposition across the Respiratory Tract

**DOI:** 10.3390/pharmaceutics12030230

**Published:** 2020-03-05

**Authors:** Lihi Shachar-Berman, Saurabh Bhardwaj, Yan Ostrovski, Prashant Das, Pantelis Koullapis, Stavros Kassinos, Josué Sznitman

**Affiliations:** 1 Department of Biomedical Engineering, Technion—Israel Institute of Technology, Haifa 3200003, Israel; lihi.shachar@gmail.com (L.S.-B.); saurabh@campus.technion.ac.il (S.B.); yanost@gmail.com (Y.O.); 2 Department of Mechanical Engineering, University of Alberta, Edmonton, AB T6G 2R3, Canada; pdas@ualberta.ca; 3 Computational Sciences Laboratory (UCY-CompSci), Department of Mechanical and Manufacturing Engineering, University of Cyprus, Nicosia 1678, Cyprus; koullapis.g.pantelis@ucy.ac.cy (P.K.); kassinos@ucy.ac.cy (S.K.)

**Keywords:** in silico simulations, computational fluid dynamics, inhalation therapy, aerosols, fibers, respiratory tract

## Abstract

Motivated by a desire to uncover new opportunities for designing the size and shape of fiber-shaped aerosols towards improved pulmonary drug delivery deposition outcomes, we explore the transport and deposition characteristics of fibers under physiologically inspired inhalation conditions in silico, mimicking a dry powder inhaler (DPI) maneuver in adult lung models. Here, using computational fluid dynamics (CFD) simulations, we resolve the transient translational and rotational motion of inhaled micron-sized ellipsoid particles under the influence of aerodynamic (i.e., drag, lift) and gravitational forces in a respiratory tract model spanning the first seven bifurcating generations (i.e., from the mouth to upper airways), coupled to a more distal airway model representing nine generations of the mid-bronchial tree. Aerosol deposition efficiencies are quantified as a function of the equivalent diameter (*d_p_*) and geometrical aspect ratio (*AR*), and these are compared to outcomes with traditional spherical particles of equivalent mass. Our results help elucidate how deposition patterns are intimately coupled to *d_p_* and *AR*, whereby high *AR* fibers in the narrow range of *d_p_* = 6–7 µm yield the highest deposition efficiency for targeting the upper- and mid-bronchi, whereas fibers in the range of *d_p_*= 4–6 µm are anticipated to cross through the conducting regions and reach the deeper lung regions. Our efforts underscore previously uncovered opportunities to design the shape and size of fiber-like aerosols towards targeted pulmonary drug delivery with increased deposition efficiencies, in particular by leveraging their large payloads for deep lung deposition.

## 1. Introduction

Inhalation therapy is an attractive drug delivery method for both topical and systemic treatments, the advantages of which include avoiding injections and circumventing the toxicity of the digestive system, which in many cases degrades the drug [1,2]. In general, however, only a mere 10–15% of an inhaled drug typically deposits in the targeted regions of the lungs, whether it is the bronchial regions or the deep acinus [3,4,5]. While the majority of larger micron-sized spherical aerosols deposit in the extra-thoracic airways (spanning from the mouth to the throat) under the effects of impaction [3], fiber-shaped aerosols have the ability to follow airflow streamlines, thereby offering an opportunity to bypass such screening and potentially reach deeper into the lungs [6]. Once these fibrous particles deposit in the deep lung parenchyma, phagocytosis may be inhibited, as macrophages undergo significant distortion when trying to engulf the elongated particles [7,8,9,10]. Considering that fibers may carry a higher therapeutic payload due to their relatively larger surface area compared to spheres of equivalent volume, these geometrical properties may offer advantages in conceiving attractive carriers for pulmonary therapeutic delivery. 

A number of previous works have experimentally examined various deposition characteristics of fibers in upper airways. For example, Chen et al. [11] numerically and experimentally investigated fiber deposition in a single bifurcating airway model. Wang et al. [12] investigated the humidity effect (15% Relative Humidity (RH)) on glass fibers and focused on the electrostatic forces that highly affect deposition outcomes. In the following study, Wang et al. [13] characterized deposition patterns of glass fibers in realistic human nasal airways according to the particle geometry and flow rate. More recently, Belka et al. [14] showed the lower deposition efficiency of glass fibers compared to spherical particles in an upper airway model spanning the oral cavity to the seventh bronchial generation, underlining a potential to leverage fiber-shaped carriers to bypass deposition across the upper airways. In this context, several trials modified therapeutic carriers into elongated shapes. Among them are the works of Zheng et al. [15], who modified lactose carriers, and Ikegami et al. [16], who modified steroids that are widely used for inhalation therapy. However, selecting the optimal shape and size for augmented deposition characteristics has remained widely elusive.

Given the challenges of running large parametric studies experimentally, numerical studies using computational fluid dynamics (CFD) have supported investigations into the transport and deposition patterns of fibrous particles along the respiratory tract. Among them, Dastan et al. [17] suggested an equivalent diameter and impaction parameter to characterize deposition of fibers in nasal airways. Feng and Kleinstreuer [18] simulated micron particles with different aspect ratios (*AR*) in an upper airway model, at a fixed equivalent diameter of 2.16 μm. The authors showed that such fibers are screened through the model as *AR* increases, and are therefore potentially more harmful as they pass through the upper airways and enter the deeper lungs. Holmstedt et al. [19] investigated the dynamics and deposition patterns of oblate and prolate spheroids spanning the nano- and micro-scales but were limited by considering only a straight tube model of a bronchial duct. Notably, in silico studies to date have been limited to very narrow windows of particle sizes (if not a fixed size), oftentimes overlooking the potential of larger micron-sized aerosols that are typically screened when spherical. 

Recently, we began exploring fiber deposition characteristics in small acinar domain models in a bottom-up approach [2], where fibers in the range of 4–6 μm were identified as potentially attractive sizes for deposition in the deep lung regions. Motivated by such preliminary outcomes, in the present work we explore opportunities to design fiber-like particles in the context of dry powder inhalation (DPI), whereby leveraging their aerodynamic properties can be advantageous in crossing through the conductive airways and ultimately reaching the deeper lungs. In particular, we identify windows for optimal geometrical characteristics of such fibers (i.e., size and shape) to target deep lung regions, such as the bronchioles and the acinus, by avoiding both sedimentation and interception. Such in silico results may help open avenues for manufacturing specific powders with particulate matter shapes and sizes that can ultimately improve inhalation therapy in both distal lung pathologies (i.e, pneumonia, cystic fibrosis, emphysema) and systemic delivery of drugs and vaccines through the alveolar capillaries into the cardiovascular system [20,21,22].

## 2. Numerical Procedures

### 2.1. Airway Geometry and Inhalation Conditions

**Figure 1:**
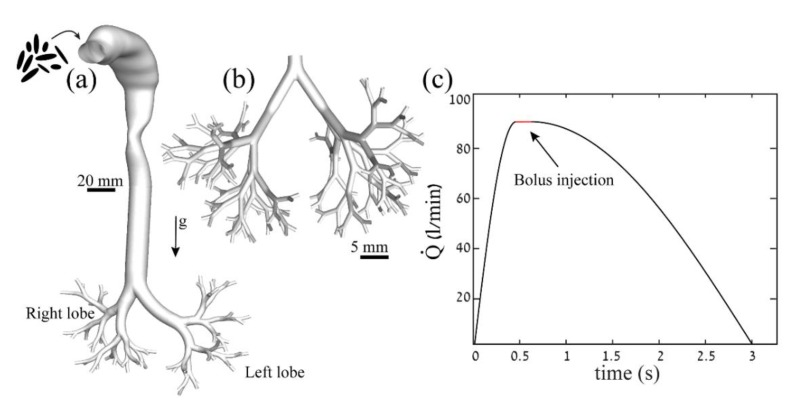
Schematic of the lung domains and dry powder inhaler (DPI) flow profile. (**a**) The in silico upper airways model consists of a mouth and throat, trachea, and 7 generations of the conducting airways, following [25]. (**b**) The bronchial tree model consists of 9 symmetrical generations of the bronchial airways, following [23]. (**c**) Profile of the DPI inhalation maneuver (i.e., flow rate) through the mouth as a function of time. Particle injection (marked in red) is confined to a short bolus, spanning 0.45 to 0.6 s [25].

As recently highlighted [23,24], spatially and temporally resolving airflow and aerosol transport dynamics across a complete lung model with vast multiscale properties spanning over 20 airway bifurcations of the pulmonary tree requires high computational resources that are still typically beyond reach. In turn, our strategy revolves around in silico numerical simulations of ellipsoid-shaped fibers of varying equivalent diameters (*d_p_*) and aspect ratios (*AR*) in an upper airways model and a bronchial tree, adopting a multiscale approach in the footsteps of Koullapis et al. [23]. In particular, the upper airways domain (see Figure 1a) follows recent work [25] on the fate of inhaled spherical aerosols in upper airway models (i.e., from the mouth to the 6th generation of the tracheobronchial respiratory tree). The mouth–throat region is modelled following the work of [26], and the first seven generations of the bronchial airway tree are modelled according to the seminal morphometric studies [27,28]. The following nine-generation bronchial tree (see Figure 1b) was constructed as idealized dichotomous bifurcations that match a physiologically realistic model [29]. Briefly, the bronchial airways range from 2.45 mm down to 0.5 mm in diameter (i.e., generations 7 to 16). Tetrahedral meshes were selected and generated with ANSYS ICEM-CFD commercial software following a rigorous mesh independence and sensitivity study (see Figures S1–S3 in Supplementary Materials). A final mesh containing 2,238,134 nodes and 6,156,103 elements was generated for the upper airway model and a mesh containing 1,729,084 nodes and 4,502,178 elements was generated for the bronchial tree. Here, we consider a simulated breathing maneuver that mimics a DPI profile (see Figure 1c) as a potential method to deliver non-spherical particles as a dry powder. The tidal volume (TV) is estimated as 2.95 L, the peak inspiration flow rate (PIFR) is estimated as 90 L/min, and the total inspiration period is 3 s [25] for an average human adult. 

### 2.2. Airflow Simulations and Boundary Conditions

The motion of air is governed by the continuity and momentum (i.e., Navier–Stokes (N–S)) equations. The ANSYS Fluent (ANSYS, Inc., Canonsburg, PA, USA) commercial software was used to perform the transient flow simulations, in which the mass and momentum (i.e., Navier–Stokes) conservation equations are solved numerically. It is well known that airflows in the respiratory upper airways involve laminar, transitional, and turbulent flow regimes [25,30,31], with the Reynolds number (Re) in the extra-thoracic regions ranging between several hundred to over 5000 during heavy breathing. For the flow scenarios described in the upper airways domain, we selected the realizable *k-ε* viscous model to solve the N–S equations [32], with a final time step of 0.01 s. This model is one of the two-equation models in the group of Reynolds-Averaged Navier–Stokes (RANS)-based turbulence models, and its advantage over the classic *k-ε* model lies in providing improved predictions for the spreading rate of both planar and round jets (e.g., in the case of a laryngeal jet [25,33]). The realizable *k*-*ε* model is essentially an extension of the classic *k*-*ε* model that inherits all the standard features of the classic *k*-*ε* model. Not only does the realizable *k*-*ε* model capture transient characteristics in turbulent flows, the model exhibits good performances in capturing strong pressure gradients, flow separation, and recirculation characteristics [34]. The mathematical form of governing equations for fluid flow (i.e., N–S equations and the realizable *k-ε* model [35,36]) are provided in the Supplementary Materials. Here, we consider unsteady flows resulting from the specific inhalation profile (Figure 1c), as the rotational movements of fibers are known to be highly sensitive to transient changes in flow rate and ensuing local velocity gradients [2,37]. A Semi-Implicit Method for Pressure Linked Equations-Consistent (SIMPLEC) scheme of a pressure–velocity coupling solution method was implemented to achieve improved and faster convergence, with a least squares cell-based approach used for the gradient spatial discretization. A second-order scheme was chosen for pressure, whereas a second-order upwind scheme was selected for momentum, turbulent kinetic energy, and turbulent dissipation rate for higher accuracy. Such algorithmic schemes have been thoroughly implemented in previous studies, exhibiting similar flow physics [25]. 

In parallel, following our multiscale approach, the bronchial model spanning generations 7 to 16 is simulated separately due to the different range of Re values across the airway tree, which decreases from approximately ~10^3^ at peak inhalation around the larynx constriction to ~10^2^ at the entrance of the lower bronchial tree [23,31,38]. Therefore, a viscous laminar model was implemented with a SIMPLE scheme of pressure–velocity coupling to describe the laminar flow that characterizes this region. A least squares cell-based approach was defined for the gradient spatial discretization, a second-order scheme was chosen for pressure, and a second-order upwind scheme was chosen for momentum solutions [25]. 

A velocity inlet is defined at the mouth opening (Figure 1a) following the DPI inhalation flow rate described in Figure 1c. Along the airway walls, a “no slip” condition is imposed and the outlets are defined according to physiologically realistic ventilation distributions into each lung lobe [25,39,40,41]; namely, 15% to the left upper lobe, 31% to the left lower lobe, 14% to the right upper lobe, 7% to the right middle lobe, and 33% to the right lower lobe. Specifically, outflow conditions are weighted to ensure that the mass flow distribution through each lobe corresponds to established physiological estimates [41]. Here, the fractional flow rate boundary condition is implemented in ANSYS Fluent. Briefly, this signifies that pressure at the outlets is not directly specified, but rather values are determined by the solver using the upstream flow conditions, where the “flow rate weighting” option is used to specify the percentage of the inlet flow that is distributed to each lung lobe [25]. The imposed flow rate profile for DPI (Figure 1c) is scaled by the average flow that exits the outlets of the upper airways model and is defined at the inlet of the bronchial tree (i.e., the scaling factor defined by the ratio of the averaged velocity at the inlet of each domain). Note that all airway walls across the domains obey a “no slip” condition and the outlets are defined as equally dividing the flow. 

### 2.3. Fiber Dynamics

The discrete element method (DEM) algorithm, which was validated in a recent study [37], is implemented within the commercial solver (ANSYS Fluent) as a user-defined function (UDF) [2,37,42] with a time step of $2\times 10^{-7}$ s to simulate the motion of particles. Ellipsoid-shaped fibers with a density of 1000 kg/m^3^ and a mass (i.e., volume) equivalent to spheres spanning a size range of 1–20 µm are simulated [23,25]. This broad size range of fibers is selected as such particles are known to have the ability to deposit across the lungs [43]. The aspect ratio of our simulated fibers spans *AR* ≈ 1 (representing a sphere) to 30. A detailed description of particle characteristics is reported in the Supplementary Materials (see Figures S4–S5), where $a_{p}\;$and $b_{p}$ are the minor and major axes of the fiber, respectively; $d_{\mathrm{Stk}}$ is the fiber equivalent volume diameter given by [44], with $d_{\mathrm{Stk}}=2a_{p}\sqrt{\frac{AR\ln\left(AR+\sqrt{AR^{2}-1}\right)}{\sqrt{AR^{2}-1}}}$. Here, $t_{0}$ is the relaxation time, calculated as $t_{0}=\frac{\rho_{p}d_{\mathrm{Stk}}{}^{2}}{18\mu_{air}}$, $\mu_{air}$ is the dynamic viscosity of air, and Stk is the particle Stokes’ number for fibers, calculated as $\mathrm{Stk}=t_{0}u_{0}/D$, where $u_{0}$ is the maximal velocity in the inlet of each domain during peak inhalation and D is the inlet diameter in each of the domains. Note that as *AR* increases, $d_{\mathrm{Stk}}$ decreases, along with the relaxation time and Stk; in turn, such particles reach equilibrium faster with the surrounding flow field (i.e., acting as flow tracers). The transport dynamics of micron-sized airborne particles are foremost governed by viscous drag, aerodynamic lift, and gravity (operating in the negative *y*-direction in upper airways, and in the positive *z*-direction in the bronchi model). Concurrently, for such particle diameters, Brownian motion may be neglected—the transport mechanism most relevant for sub-micron particles [45]. Other forces, such as hygroscopic growth and electrostatic effects, are effectively neglected. We implement this Euler–Lagrangian model with drag and lift forces, which are time- and space-dependent, according to the particle orientation and the surrounding velocity gradients of the flow field. 

Thirty-six individual groups of combinations of *d_p_* and *AR* (see Table S1 in Supplementary Materials), representing a total of 108,000 particles, are introduced as a uniform Cartesian mesh mapped onto the inlet of the domain, namely at the mouth opening (Figure 1a). Table S2 summarizes the various flow and particle properties, along with all computational details. Subsequently, the particle bolus entering the bronchial tree is delayed according to the finite time it takes the flux to fill the upper airway domain and reach the 7th generation (Figure 1c). Convergence tests were carried out for the number of particles simulated (see Figure S6 in Supplementary Materials). Particles are initially positioned parallel to the streamwise flow, as they are assumed to have been subjected to high moments that immediately lead to alignment with the flow [18]. Deposition mechanisms of inhaled fibers include inertial impaction and sedimentation. In stark contrast to spheres, interception becomes a significant mechanism of deposition [46,47], and is, thus, incorporated in the simulations. We recall that interception occurs when the center of mass of a fiber-shaped particle stays in a fluid streamline, however due to its elongated profile, the particle tip comes into physical contact with the airway’s surface. We assume that any contact with the airway wall (i.e., entrapment by airway mucus) results in deposition.

## 3. Results and Discussion

Previous in silico studies of fibers in upper airways have either investigated specifically sized groups of particles or a limited anatomical airway model; moreover, studies have been mostly limited to steady inhalation flow conditions [18,48]. In an effort to go beyond past investigations and uncover opportunities for drug carrier shape design, we specifically explore here the coupled effect of *AR* and $d_{p}$ on the fate of fibers inhaled into the respiratory system up to generation 15 under DPI flow conditions [23,25]. We present deposition characteristics of non-spherical particles characterized by nine equivalent spherical diameters (i.e., 1–7, 10, and 20 μm) and four distinct values of *AR* (i.e., 1, 3, 10, and 30) in an anatomically inspired upper airway model and a symmetric nine-generation bronchial tree. To gain some initial insight, we begin by giving a qualitative overview of the deposition patterns of such fibers.

### 3.1. Deposition Patterns of Fibers in the Conducting Regions

Micron-sized particles are known to deposit in large airways (e.g., nasal, oral, and upper tracheobronchial airways) during inhalation, mainly due to impaction, secondary flow convection, and turbulent dispersion [31]. Thus, enhanced particle deposition appears at stagnation points, where particles cross stream lines and disrupt axial motion. Here, we recall three major deposition hot spots. The first one is the outer bend of the larynx, immediately upstream of the constriction. With a steep geometry change, particles tend to impact the airway wall following direct impaction and secondary flows. The second spot can mostly be observed directly after the constriction, due to inertial impaction arising from the laryngeal jet and secondary motion [31,49]. The third occurs around the carinal ridges along the gravity direction, due to inertial impaction as well as sedimentation. Another important parameter is the ability of drug particles to disperse. Dispersion of aerosols enables both effective respiratory delivery [50] and reduced toxicity effects, due to high local deposition concentration of hazardous particles [45,51,52]. 

**Figure 2:**
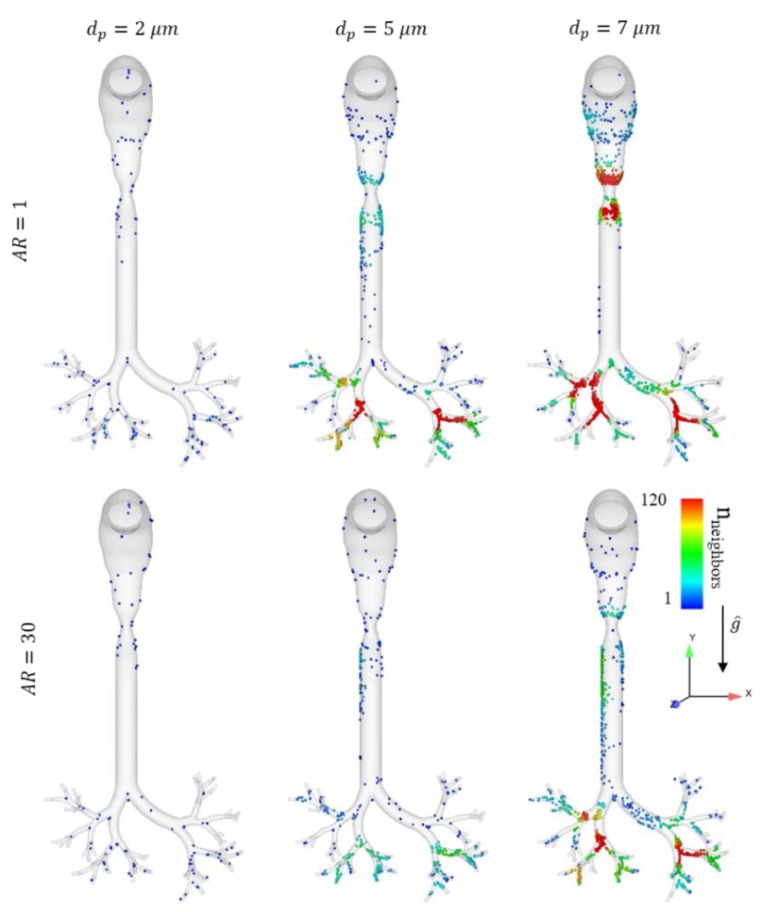
Deposition maps for fibers with selected aspect ratios (*AR*; rows) and equivalent diameters (columns) at the end of the DPI maneuver (*t* = 3 s) in the upper airway domain. The particle deposition is color coded according to the number of neighbors within a 10 mm radius. It is clear that fibers of equivalnt diameter (*d_p_*) > 2 mm deposit less and disperse more compared to spheres. We note that 7 µm particles are strongly influenced by impaction, causing them to be deposited in the characteristic “hot spots” of the domain, although elongation (i.e., the *AR*) qualitatively increases dispersion outcomes.

**Figure 3:**
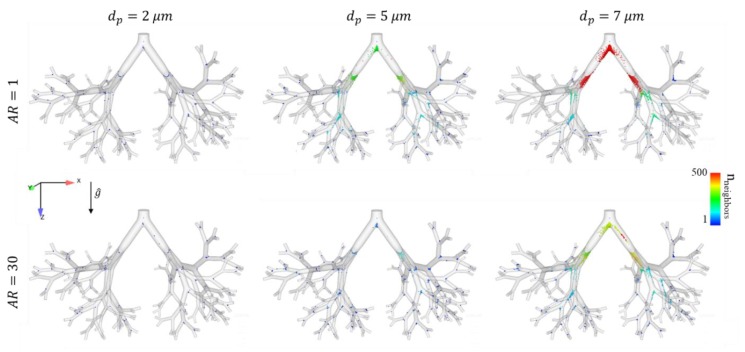
Deposition maps for fibers with different aspect ratios (rows) and equivalent diameters (columns) at the end of the DPI maneuver (*t* = 3 s) in the nine-generation bronchial tree domain. Particle deposition is color coded (1–500) according to the number of neighbors within a 5 mm radius. It is clear that particles of *d_p_* > 2 µm fibers deposit less and disperse more compared to spheres. We note that as *d_p_* increases, particles are strongly influenced by impaction and sedimentation at the carinas, causing them to be deposited, although they are more dispersed as the *AR* increases.

Figure 2 presents deposition maps in upper airways at the end of the DPI maneuver (*t* = 3 s) for six distinct particle groups. We compare the traditional so-called “best choice” (i.e., typically 2 μm spheres) with equivalent fibers (*AR* = 30) of the same volume, as well as 5 and 7 μm spheres and fibers for further evaluation and comparison. The color coding represents the dispersion index of the deposited particles, as estimated by calculating the number of neighbors within a given neighborhood (i.e., 10 mm radius) [42]. In general, our in silico results suggest that particle deposition increases with *d_p_*, as the roles of impaction and sedimentation become more significant during airborne flight; this quantitatively translates into an increase in the number of nearest neighbors upon deposition. It is qualitatively observed that 2 μm spheres are deposited similarly, as fibers of the same volume and most of the particles of this size group are screened through the model. As $d_{p}$ increases, we qualitatively note that sedimentation becomes more dominant, whereas 5 μm spheres are much less dispersed compared to 5 μm fibers, and are deposited with much higher efficiency. The 7 μm spheres are densely deposited around the larynx constriction, whereas the 7 μm fibers are dispersed significantly better and are only densely deposited at the second bifurcation of the bronchi. Figure 3 presents similar deposition patterns of the particles assembled in a 9-generation bronchial tree at the end of the DPI maneuver (*t* = 3 s), as most of the 2 μm particles are screened through the model, without a noticeable difference between spheres and fibers. Conversely, the 5–7 μm particles are deposited mainly at the carinas, and the difference between fibers and spheres is significant in terms of deposition and dispersion, as fibers are able to follow the streamlines out of the model and are generally more dispersed. 

### 3.2. Deposition Efficiencies

**Figure 4:**
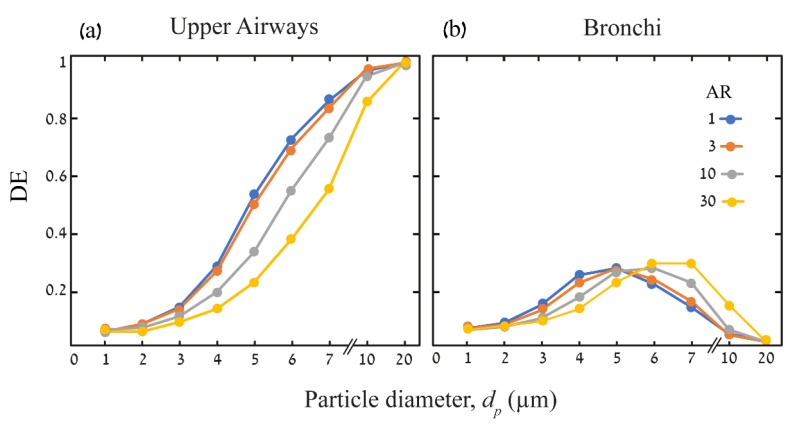
Deposition efficiencies (DE) in upper airways model (**a**) and the bronchial bifurcating tree (**b**) as a function of *d_p_* and *AR* at the end of the DPI maneuver (*t* = 3 s). (**a**) As *d_p_* increases, particles deposit more and a significant effect of AR only appears for particles larger than 4 µm; as *AR* increases, DE decreases. For 20 µm particles, DE ~ 1 without observable differences between spheres and fibers. (**b**) DE in the bronchial tree is calculated by reducing the particles that already deposit in upper airways; therefore, an optimum is achieved for deposition in the bronchi. While small particles (*d_p_* < 3 µm) are screened through the domain due to convection, large particles (*d_p_* =10–20 µm) deposit in the upper airways due to inertial impaction and gravitational sedimentation. The optimum for particles deposition in the bronchi changes with *AR*, such that 5 µm spheres reach DE ~ 0.26 and 7 µm fibers of *AR =* 30 reach DE ~ 0.3.

Figure 4 summarizes the ensuing deposition efficiencies (DE) in the upper airways and the bronchial tree at the end of the DPI maneuver (*t* = 3 s). Here, our in silico results suggest that the DE of particles in the upper airways increase monotonically with *d_p_*. However, as *AR* increases, particles are screened through the first seven generations and enter into the bronchial tree, thereby translating into a lower DE. For *d_p_* > 20 μm, all particles are deposited, regardless of the *AR.* We recall that in the nine-generation bronchial tree, DE is calculated after reducing the particles that have already been deposited in the upper airways, such that deposition outcomes appear to be an optimum achieved between convection of small particles (*d_p_* < 4 μm) to impaction and sedimentation of large particles (*d_p_* > 7 μm). Here, an increase in *AR* is seen to shift the optimum from *d_p_* = 5 μm for spheres to *d_p_* = 7 μm for fibers of *AR* = 30. 

In view of such results, and within the specific modelling conditions conducted here, we observe that 6–7 μm fibers have the potential to target the upper airways of the bronchial region. In addition, 4–6 μm fibers appear to be able to penetrate through the conducting region and continue to lower lung regions, and thus hypothetically enter the deep acinar regions. In a previous numerical study of fiber deposition in the acinar region [2], we demonstrated that while 1–3 μm fibers have the ability to penetrate deep into the lungs, these are likely to be exhaled due to the high convection resulting from their aerodynamic properties, which leads to lower deposition. Therefore, we suggest that 4–6 μm fibers are potentially an attractive if not optimal size and shape for targeting the acinar regions of the lungs. We note that our results are indeed consistent with previous studies, whereby our results of the upper airways deposition patterns are validated against the work of [48] and [18] for the specific size range of fibers they investigated (Supplementary Materials). Furthermore, the results for spheres of different *d_p_* deposited within the bronchi are validated against the recent work of [23] (Supplementary Materials).

We compare our results with some past works that have focused on upper airway models only [48], where the authors explored a more restricted range of fiber geometries (see Figure S7 in Supplementary Materials). For example, we find that for *d_p_* = 2 μm, the deposition efficiency is less than 10% for *AR* = 30. However, for *d_p_* = 3.66 μm, the deposition efficiency is increased from 10% to 22% for the aspect ratio *AR* = 1–30, which is also found in the work of Tian and Ahmadi [48]. Similarly, our results are also consistent with the results of Feng and Kleinstreuer [18] for certain aspect ratios, specifically *AR* = 3 and 10 (see Figure S8 in Supplementary Materials). We further compared the present results in the bronchial region for spherical particles with the recent work of Koullapis et al. [23]. Generally, our results are in good agreement for particle diameters of *d_p_* = 1–2 μm and 2–5 μm (*AR* = 1–30), as shown in Figure S9 (see Supplementary Materials). The deposition efficiency in this range during the inhalation phase is found to be ~5% for *d_p_* = 1–2 μm and ~20% for *d_p_* = 2–5 μm. It is worth briefly mentioning that upon analyzing our deposition results with the assumption of an equivalent diameter analogy, following $d_{\mathrm{Stk}}$ as the fiber equivalent volume diameter [44], we find that the deposition efficiency may be predicted for a narrow range of fiber *AR*, which could help reduce computational efforts for a full-scale fiber simulation. Indeed, this assumption would be useful for particle diameters ≤3 μm and ≥10 μm, where we can achieve identical deposition efficiency with the equivalent diameter method compared to full scale fiber simulations. In the latter case (≥10 μm), sedimentation is likely to govern deposition outcomes, such that resolving the complex transient dynamics of fibers is unnecessary. Nevertheless, for the range of fibers with diameters of 3–10 μm that are of specific interest in the present study following recent work [2], this analogy comes short of capturing the complex transport and deposition dynamics of such fibers. 

We briefly comment on some of the limitations of the present numerical study. To begin, our upper airways model is based on a generic anatomy [26] and seminal morphometric studies [25,27,28], while anatomical differences between subjects will undeniably affect deposition outcomes, and thereby modulate the optimal size and shape window in augmenting targeting efficiency of fibers (i.e., combination of *d_p_* and *AR*). Moreover, we do not model the cartilaginous rings that are present in the trachea and upper bronchi. These rings are essential for airway stabilization, forming an uneven surface that may influence air flow and particle deposition [53,54]. Our boundary conditions for the outflow simply mimic approximate flow distributions, while there are more realistic approaches to define the outflow, such as in recent work [55], where resistance and compliance measurements of the outflow were derived from experiments and implemented in the outlets of the upper airways of a healthy and an emphysematic rat lung. It is common to assume that the complex flows developed in the oral airways subsequently propagate into the tracheobronchial airways. Hence, the realistic inlet conditions obtained from the upper head airways should be applied to the bronchial airways, rather than scaling the DPI profile to the 7th generation inlet. 

Within our bronchi model, the direction of gravity is fixed, whereas in reality bronchial trees surrounding the upper airways are oriented across all spatial directions, which may affect deposition efficiencies, specifically in the size range of 5–10 μm particles. Furthermore, deposition during exhalation is not included in our results, while recent work [23] showed that 10–15% of particles inhaled in the size range of 2–10 μm potentially deposit during exhalation. Another limitation to our simulations may stem from neglecting particle–particle interactions as agglomeration, which might be mostly relevant in upper airways. Here, we have considered one-way coupling between the particles and the flow; thereby, local effects of long fibers on airflows in the surrounding areas of curved surfaces are not included in calculating the airflow patterns. Despite such limitations, the general trends resulting in the present study are anticipated to hold true. In particular, while often overlooked in the past, our findings highlight the existence of a window of opportunity to design fiber-shaped drug delivery carriers that will allow maximization of transport across the respiratory tract.

## 4. Conclusions

In this study, we investigated deposition patterns of fibers with different *AR* and *d_p_* values in the conducting region of the human respiratory system using in silico CFD methods. We simulated a broad parameter space of elongated particles in an anatomically faithful airway geometry under a DPI inhalation profile. Here, we suggest the existence of optimal fiber carriers for targeting deep regions of the lungs, such as the bronchioles and the acinar regions. Under the conditions investigated here, 5–7 μm fibers with high *AR* are observed to be optimal for deposition in the bronchioles, and 4–6 μm fibers with high *AR* achieve the highest deposition in the acinar regions. Interestingly, elongating the most common spherical therapeutic particles at a size of 2 μm [3] may result in lower DE in the deep regions of the lungs, due to the high convection that causes exhalation of such inhaled particles. Nevertheless, aiming for elongation of the bigger particles in a size range that is otherwise known to deposit mostly along the mouth–throat region may improve DE in the deep regions of the lungs, as those particles deposit due to sedimentation and interception (both significant during flow reversal in breathing). For those fiber-shaped particles that are likely to be screened and are anticipated to deposit across the upper airways and conducting regions of the lungs, where mucociliary clearance is active, it is acknowledged that ensuing clearance rates will likely be independent of particle shape and size (as well as charge), as supported by previous ex vivo and in silico studies [56,57]. While there is still a gap to bridge in order to translate our results into recommendations, the principles of fiber aerodynamics in determining DE in the deep regions of the lungs suggest opportunities for improvement in carrier design for inhalation therapy.

## Supplementary Materials

The following are available online at https://www.mdpi.com/1999-4923/12/3/230/s1, Figure S1. Flow profile at the larynx at pick inhalation of different mesh sizes. Figure S2. Velocity profiles at the center line (left) and at the first bifurcation (right) of 3 different mesh densities. Figure S3. Flow patterns at the first bifurcation of the bronchial tree in three different mesh densities. Figure S4. Definition of two auxiliary coordinate systems. Figure S5. Examples of 3 simple cases to illustrate Euler angle. Figure S6. DE results of different number of particles, shown in each plot. Negligible difference was found between 1500 and 3000 particles results. Figure S7. Validation of upper airways deposition against the work of Tian&Ahmadi [48] (shown on the left). On the right-the results of the current study match the black squares describing the results of Tian&Ahmadi [48]. Figure S8. Validation of upper airways deposition against the work of Feng&Kleinstreuer [18] (left). In black squares, the matched results of *AR* = 3,10 are shown in both plots. Figure S9. Validation of bronchial deposition against the work of Koullapis et al. [23] (left). In black squares, the matched results of spheres *dp* = 1–2 μm, *dp* = 2–5 μm are shown (right). Table S1. Particle non dimensional characteristics for all size and *AR* ensembles. Table S2. Computational details and various properties of flow and particles used in the present simulations.
