# Supplementary material for: In Silico Optimization of Fiber-Shaped Aerosols in Inhalation Therapy for Augmented Targeting and Deposition across the Respiratory Tract
Source: Pharmaceutics. 2020 Mar 5;12(3):230. doi: 10.3390/pharmaceutics12030230 (PMC7150950; doi:10.3390/pharmaceutics12030230)
Supplement: Supplementary file 1 [file pharmaceutics-12-00230-s001.pdf]

pharmaceutics

MDPI

Supplementary Materials: In Silico Optimization of Fiber-Shaped Aerosols in Inhalation Therapy for Augmented Targeting and Deposition across the Respiratory Tract

Governing Transport Equations

Here, we consider the Navier–Stokes equations describing the conservation of mass and momentum for incompressible and isothermal fluid flows.

Continuity equation:

$$\frac{\partial u_j}{\partial x_j} = 0 \tag{1}$$

Momentum equation:

$$\rho \frac{\partial u_i}{\partial t} + \rho \frac{\partial \left(u_j u_i \right)}{\partial x_j} = -\frac{\partial P}{\partial x_i} + \frac{\partial}{\partial x_i}\left[\mu \left(\frac{\partial u_i}{\partial x_j} + \frac{\partial u_j}{\partial x_i} \right) \right] + \rho g_i \tag{2}$$

where *u* is the velocity vector, ρ is the mass density, *P* is the pressure, μ is the dynamic viscosity.

RANS based Realizable *k*–ε Turbulence Model

The modeled transport equations in the realizable *k*–ε model are defined as follows [34, 35-36]:

$$\frac{\partial}{\partial t}(\rho k) + \frac{\partial}{\partial x_j}(\rho k u_j) = \frac{\partial}{\partial x_j}\left[\left(\mu + \frac{\mu_t}{\sigma_k} \right) \frac{\partial k}{\partial x_j} \right] + G_k + G_b - \rho\varepsilon - Y_M + S_k \tag{3}$$

and

$$\frac{\partial}{\partial t}(\rho \varepsilon) + \frac{\partial}{\partial x_j}(\rho \varepsilon u_j) = \frac{\partial}{\partial x_j}\left[\left(\mu + \frac{\mu_t}{\sigma_\varepsilon} \right) \frac{\partial \varepsilon}{\partial x_j} \right] + \rho C_1 S_\varepsilon - \rho C_2 \frac{\varepsilon^2}{k + \sqrt{\upsilon \varepsilon}} + C_{1\varepsilon} \frac{\varepsilon}{k} C_{3\varepsilon} G_b + S_\varepsilon \tag{4}$$

where

$$C_1 = \max\left[0.43, \frac{\eta}{\eta + 5} \right], \quad \eta = S\frac{k}{\varepsilon}, \quad S = \sqrt{2 S_{ij} S_{ij}} \tag{5}$$

In these equations, μ_t is defined as turbulent dynamic viscosity, k represents turbulent kinetic energy, ε is turbulent dissipation, G_k represents the generation of turbulence kinetic energy due to the mean velocity gradients. G_b is the generation of turbulence kinetic energy due to buoyancy. Y_M represents the contribution of the fluctuating dilatation in compressible turbulence to the overall dissipation rate. C_2 and $C_{1\varepsilon}$ are constants. σ_k and σ_ε are the turbulent Prandtl numbers or *k* and ε, respectively. S_k and S_ε are user-defined source terms.

Convergence test for the Mesh in the upper airways model

We meshed the upper airways model with 2, 3, 4.8, 6, 8 million elements, and compared the velocity profile at the larynx cut plain to the results of Das *et al.* (2018) (see Figure S1).

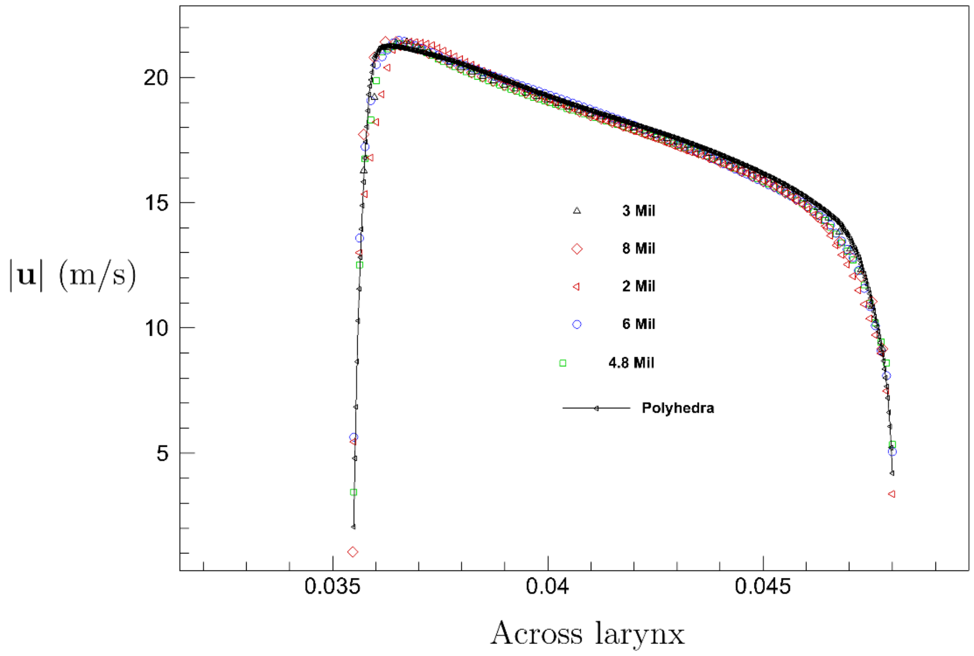

Figure S1. Flow profile at the larynx at pick inhalation of different mesh sizes.

Convergence test for the Mesh in the bronchi model

We meshed the bronchial model with 5,6.8,7.5 million elements, and compared between the velocity profile at the centreline and at the first bifurcation (see Figure S2 and Figure S3).

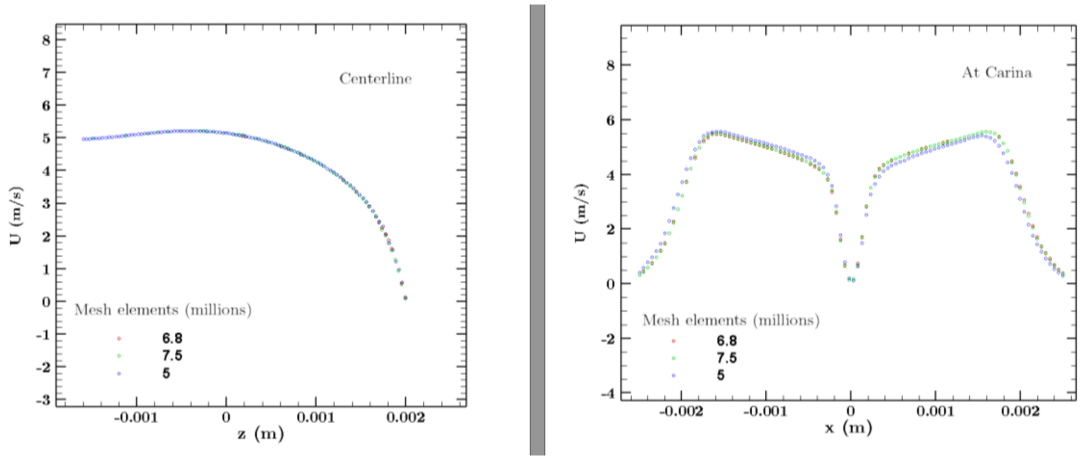

Figure S2. Velocity profiles at the center line (left) and at the first bifurcation (right) of 3 different mesh densities.

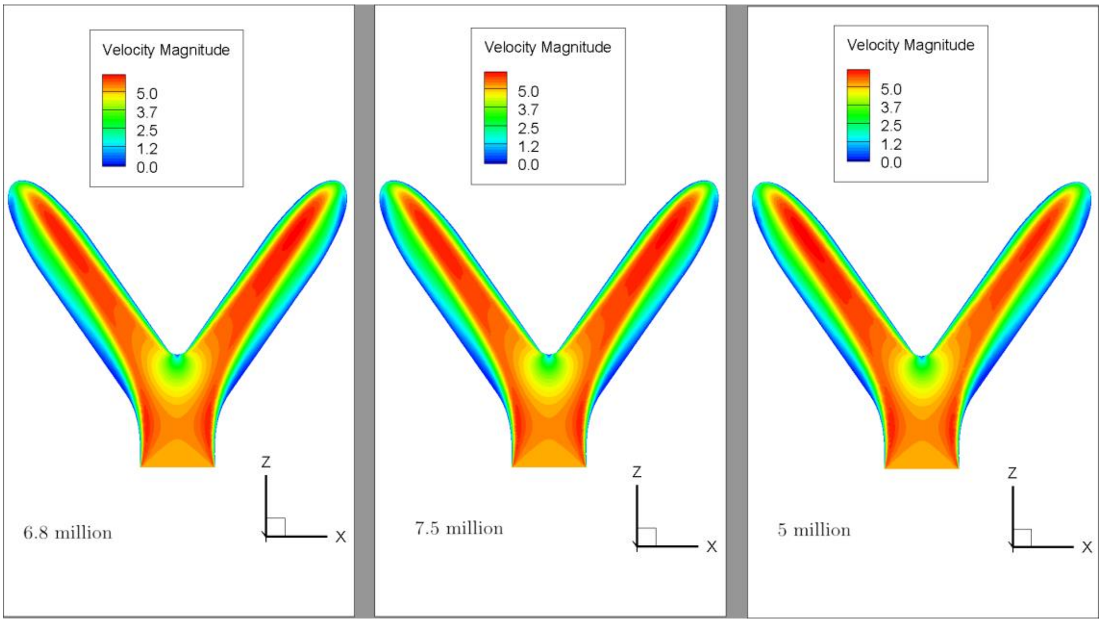

Figure S3. Flow patterns at the first bifurcation of the bronchial tree in three different mesh densities.

Ellipsoid geometric representation using Euler angles

We begin by reviewing the geometrical definitions of the particle orientation. First, let us build two additional auxiliary coordinate systems. In Figure S4, a schematic diagram of an ellipsoid is presented; we define three coordinate systems, the first (*x,y,z*), marked green, is the global lab coordinate system. The system (*x′,y′,z′*), marked black, is the particle coordinate system, its center coinciding with the particle center of mass, and the z' axis is aligned with the ellipsoid's major axis. Finally, (*x′′,y′′,z′′*), marked blue, is a coordinate system centered at the particle center of mass, but parallel to the lab coordinate system ("Euler Angles -- from Wolfram MathWorld," n.d.).

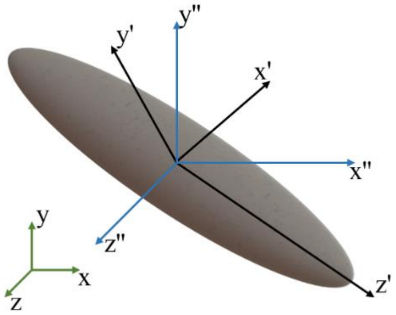

Figure S4. Definition of two auxiliary coordinate systems.

The transformation matrix between the systems (*x′,y′,z′*) and (*x′′,y′′,z′′*) is marked as 'A', such that for a vector $\mathbf{v}'$ or a matrix $\mathbf{M}'$:

$$\mathbf{v}' = \mathbf{A}\mathbf{v}'' \tag{6}$$

$$\mathbf{M}' = \mathbf{A}\mathbf{M}''\mathbf{A}^{-1} \tag{7}$$

The 'A' matrix is defined by Euler angles and Euler quaternions (Lin Tian, Ahmadi, Wang, & Hopke, 2012), as

$$\mathbf{A}=\begin{pmatrix} \cos\psi\cos\phi-\cos\theta\sin\phi\sin\psi & \cos\psi\sin\phi+\cos\theta\cos\phi\sin\psi & \sin\psi\sin\theta \\ -\sin\psi\cos\phi-\cos\theta\sin\phi\cos\psi & -\sin\psi\sin\phi+\cos\theta\cos\phi\cos\psi & \cos\psi\sin\theta \\ \sin\theta\sin\phi & -\sin\psi\cos\phi & \cos\theta \end{pmatrix} \tag{8}$$

where: we mark the vector N (orange) to be the intersection of the two planes, (x-y) with the plane (x'-y'); ϕ is the angle between x and N; θ is the angle between z to z'; ψ is the angle between x' and N

In Figure S5, we have 3 examples illustrating this idea:

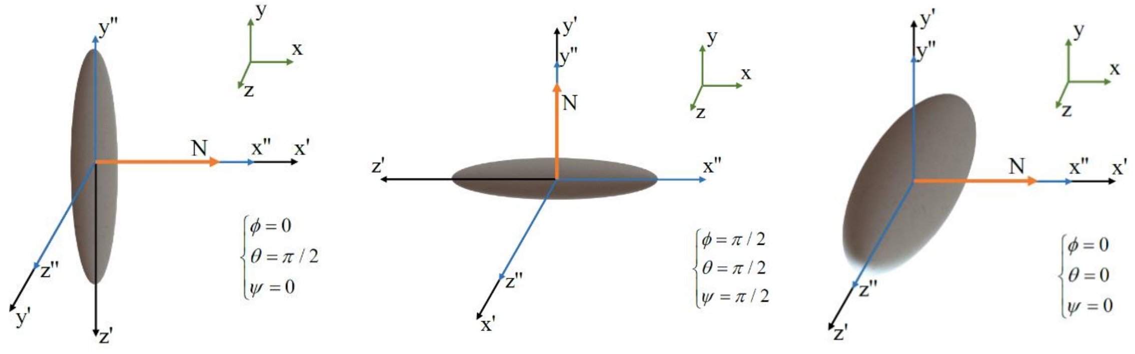

Figure S5. Examples of 3 simple cases to illustrate Euler angle.

Euler Quaternions

The use of Euler angles directly in the solution of particle's motion, will lead to singularities (Fan & Ahmadi, 1995) at $\theta=0,\pi$. Therefore, to solve this problem we use Euler quaternions to visually describe the particle position and orientation, as defined in the following formula (Feng & Kleinstreuer, 2013):

$$\begin{cases} \varepsilon_1=\cos\left(\frac{\phi}{2}-\frac{\psi}{2}\right)\cdot\sin\frac{\theta}{2} \\ \varepsilon_2=\sin\left(\frac{\phi}{2}-\frac{\psi}{2}\right)\cdot\sin\frac{\theta}{2} \\ \varepsilon_3=\sin\left(\frac{\phi}{2}+\frac{\psi}{2}\right)\cdot\cos\frac{\theta}{2} \\ \eta=\cos\left(\frac{\phi}{2}+\frac{\psi}{2}\right)\cdot\cos\frac{\theta}{2} \end{cases} \tag{9}$$

We note here that quaternions must always satisfy the condition

$$\varepsilon_1^2+\varepsilon_2^2+\varepsilon_3^2+\eta^2=1 \tag{10}$$

Finally, in terms of the Euler quaternions, the rotation matrix 'A' now reads:

$$\mathbf{A}=\begin{pmatrix} 1-2\left(\varepsilon_2^2+\varepsilon_3^2\right) & 2\left(\varepsilon_1\varepsilon_2-\varepsilon_3\eta\right) & 2\left(\varepsilon_1\varepsilon_3-\varepsilon_2\eta\right) \\ 2\left(\varepsilon_2\varepsilon_1-\varepsilon_3\eta\right) & 1-2\left(\varepsilon_3^2+\varepsilon_1^2\right) & 2\left(\varepsilon_2\varepsilon_3+\varepsilon_1\eta\right) \\ 2\left(\varepsilon_3\varepsilon_1+\varepsilon_2\eta\right) & 2\left(\varepsilon_3\varepsilon_2-\varepsilon_1\eta\right) & 1-2\left(\varepsilon_1^2+\varepsilon_2^2\right) \end{pmatrix} \tag{11}$$

For the completeness of this section, we develop a simple expression to convert the orientation of the particle represented in Euler quaternions, to a simple direction vector representation. This conversion is particularly useful when visualizing calculated behavior of such particles. We imagine the particle to be a unit vector in coordinate system (x',y',z'):

$$\mathbf{P}'=\left(0,0,1\right)^T \tag{12}$$

We remind that the z' direction is the direction of the ellipsoid's major axis. Using the transformation matrix, the rotated particle would then be

$$\mathbf{P}'' = \mathbf{A}^{-1}\mathbf{P}' = \begin{pmatrix} 2(\varepsilon_1\varepsilon_3 + \eta\varepsilon_2) \\ 2(\varepsilon_2\varepsilon_3 - \eta\varepsilon_1) \\ 2(-\varepsilon_1^2 - \varepsilon_2^2 + 0.5) \end{pmatrix} \tag{13}$$

where

$$\mathbf{A}^{-1} = \begin{pmatrix} \dots & \dots & 2(\varepsilon_1\varepsilon_3 + \eta\varepsilon_2) \\ \dots & \dots & 2(\varepsilon_2\varepsilon_3 - \eta\varepsilon_1) \\ \dots & \dots & 2(-\varepsilon_1^2 - \varepsilon_2^2 + 0.5) \end{pmatrix} \tag{14}$$

Force Balance

An ellipsoid particle, moving in a shear flow at a low particle Reynolds number is subjected to the gravitational force ($F_{gravity}$), the hydrodynamic drag force (F_{drag}) and the lift force (F_{lift}). The equation for conservation of linear momentum reads

$$m_p \frac{d\mathbf{v}}{dt} = \mathbf{F}_{gravity} + \mathbf{F}_{drag} + \mathbf{F}_{lift} \tag{15}$$

Subscripts p and f represent particle and fluid respectively. The mass an ellipsoid particle used in equation (14) is given by:

$$m_p = \frac{4}{3}\pi a_p^3 AR \rho_p \tag{16}$$

Where a_p is the particle's semi-minor axis (the semi-major axis will be marked as b_p), $AR = b_p / a_p$, and ρ_p is the mass density. The gravitational force is calculated as

$$\mathbf{F}_{gravity} = m_p \mathbf{g} = \frac{4}{3}\pi a_p^3 AR \rho_p \mathbf{g} \tag{17}$$

Hydrodynamic Drag Force

The hydrodynamic drag force is defined as

$$\mathbf{F}_{drag} = \pi a_p \mu_f \mathbf{K}''(\mathbf{v}_f - \mathbf{v}) \tag{18}$$

where μ_f is the viscosity of the fluid, and $\mathbf{K}''$ is the resistance tensor accounting for a particle's shape. The resistance tensor for an ellipsoid in the particle coordinate system, $\mathbf{K}'$, is given by:

$$\begin{cases} \mathbf{K}'_{11} = \mathbf{K}'_{22} = \dfrac{16(AR^2 - 1)}{\dfrac{(2AR^2 - 3)\cdot \ln\left(AR + \sqrt{AR^2 - 1}\right)}{\sqrt{AR^2 - 1}} + AR} \\ \mathbf{K}'_{33} = \dfrac{8(AR^2 - 1)}{\dfrac{(2AR^2 - 1)\cdot \ln\left(AR + \sqrt{AR^2 - 1}\right)}{\sqrt{AR^2 - 1}} + AR} \end{cases} \tag{19}$$

Since the particle coordinate system is also the particle principal coordinate system, this matrix is diagonal. The transformation of this matrix to the lab global system (in which the force balance is solved) is

$$\mathbf{K}" = \mathbf{K} = \mathbf{A}^{-1} \cdot \mathbf{K}' \cdot \mathbf{A} \tag{20}$$

In fact, the resistance tensor in the particle coordinate system $\mathbf{K}'$ is constant, and the drag force is actually changing due to rotation of the particle, through the rotation matrix A. Therefore, the drag force takes the final form

$$\mathbf{F}_{drag} = \pi a_p \mu_f \mathbf{A}^{-1} \cdot \mathbf{K}' \cdot \mathbf{A}"\left(\mathbf{v}_f - \mathbf{v}\right) \tag{21}$$

Particle Lift Force

We assume that the particle is small enough that a linear shear flow can be locally approximated around the particle. The lift force is then given by

$$\mathbf{F}_{lift} = \sum_{i,j=1}^{3} \mathbf{F}_{lift}\left(i,j\right), \qquad i \neq j \tag{22}$$

$$\mathbf{F}_{lift} = \pi a_p^2 \sqrt{\frac{\rho_f}{\mu_f}} \cdot \frac{\partial v_{f,i} / \partial x_j}{\sqrt{\left|\partial v_{f,i} / \partial x_j\right|}} \cdot \left(\mathbf{K} \cdot \mathbf{B}_{i,j} \cdot \mathbf{L} \cdot \mathbf{B}_{i,j}^{-1} \cdot \mathbf{K}\right)\left(\mathbf{v}_f - \mathbf{v}\right) \tag{23}$$

where $\mathbf{B}$ is the transformation matrix of velocity gradients, added here to create a simpler to implement sum representation. This matrix is given by (Yu Feng, 2013)

$$\mathbf{B} = \begin{bmatrix} 0 & \mathbf{B}_{1,2} & \mathbf{B}_{1,3} \\ \mathbf{B}_{2,1} & 0 & \mathbf{B}_{2,3} \\ \mathbf{B}_{3,1} & \mathbf{B}_{3,2} & 0 \end{bmatrix} = \begin{bmatrix} 0 & \begin{pmatrix} 1 & 0 & 0 \\ 0 & 1 & 0 \\ 0 & 0 & 1 \end{pmatrix} & \begin{pmatrix} 1 & 0 & 0 \\ 0 & 0 & -1 \\ 0 & 1 & 0 \end{pmatrix} \\ \begin{pmatrix} 0 & 0 & 1 \\ 1 & 0 & 0 \\ 0 & -1 & 0 \end{pmatrix} & 0 & \begin{pmatrix} 0 & 0 & 1 \\ 1 & 0 & 0 \\ 0 & 1 & 0 \end{pmatrix} \\ \begin{pmatrix} 0 & 1 & 0 \\ 0 & 0 & 1 \\ 1 & 0 & 0 \end{pmatrix} & \begin{pmatrix} 0 & 0 & -1 \\ 0 & 1 & 0 \\ 1 & 0 & 0 \end{pmatrix} & 0 \end{bmatrix} \tag{24}$$

and the $\mathbf{L}$ matrix is (Harper & Chang, 1968)

$$\mathbf{L} = \begin{pmatrix} 0.0501 & 0.0329 & 0.00 \\ 0.0182 & 0.0173 & 0.00 \\ 0.00 & 0.00 & 0.0373 \end{pmatrix} \tag{25}$$

Torque Balance

Previously, the importance of solving the orientation of the ellipsoid fiber in order to solve the force balance was described. Thus, it is of special importance to solve the torque equation of this particle as well. The Euler rotation equations, i.e. the balance of torques, reads

$$\begin{cases} I_{x'} \dfrac{d\omega_{x'}}{dt} - \omega_{y'}\omega_{z'}\left(I_{y'} - I_{z'}\right) = T_{x'} \\ I_{y'} \dfrac{d\omega_{y'}}{dt} - \omega_{z'}\omega_{x'}\left(I_{z'} - I_{x'}\right) = T_{y'} \\ I_{z'} \dfrac{d\omega_{z'}}{dt} - \omega_{x'}\omega_{y'}\left(I_{x'} - I_{y'}\right) = T_{z'} \end{cases} \tag{26}$$

We note that all values here are in the particle coordinate system (x',y',z'). $\boldsymbol{\omega}$ is the angular velocity, and $\mathbf{T}$ is the torque. The principal mass moment of inertia $\left(I_{x'}, I_{y'}, I_{z'}\right)$ for an ellipsoid is given by

$$\begin{cases} I_{x'} = I_{y'} = \dfrac{\left(1+AR^2\right)}{5} a_p^2 m_p \\ I_{z'} = \dfrac{2}{5} a_p^2 m_p \end{cases} \tag{27}$$

Hydrodynamic Torque

Under the assumption that the particle is sufficiently small, and the flow around it can be approximated as a linear shear flow, the hydrodynamic torques read

$$\begin{cases} T_{x'} = \dfrac{16\pi a_p^3 AR\mu_f}{3\left(\beta_0 + AR^2\gamma_0\right)}\left[\left(1-AR^2\right)D_{z'y'} + \left(1+AR^2\right)\left(W_{z'y'} - \omega_{x'}\right)\right] \\ T_{y'} = \dfrac{16\pi a_p^3 AR\mu_f}{3\left(\alpha_0 + AR^2\gamma_0\right)}\left[\left(AR^2-1\right)D_{x'z'} + \left(1+AR^2\right)\left(W_{x'z'} - \omega_{y'}\right)\right] \\ T_{x'} = \dfrac{32\pi a_p^3 AR\mu_f}{3\left(\alpha_0 + \beta_0\right)}\left[W_{y'x'} - \omega_{z'}\right] \end{cases} \tag{28}$$

where $\mathbf{D}$ is the deformation tensor, and $\mathbf{W}$ is the spin tensor

$$\begin{cases} [\mathbf{D}]_{x'y'z'} = \dfrac{1}{2}\left(\nabla \mathbf{v}_f\right)_{x'y'z'} + \dfrac{1}{2}\left(\left(\nabla \mathbf{v}_f\right)_{x'y'z'}\right)^T \\ [\mathbf{W}]_{x'y'z'} = \dfrac{1}{2}\left(\nabla \mathbf{v}_f\right)_{x'y'z'} - \dfrac{1}{2}\left(\left(\nabla \mathbf{v}_f\right)_{x'y'z'}\right)^T \end{cases} \tag{29}$$

In Eq. (29), the fluid velocity gradient matrix is in the reference frame of the particle, i.e. it needs to be rotated from the lab frame. This can be done using Eq. (7):

$$\left(\nabla \mathbf{v}_f\right)_{x'y'z'} = \mathbf{A}\left(\nabla \mathbf{v}_f\right)_{xyz} \mathbf{A}^{-1} \tag{30}$$

Once the torques are solved (Eq. (26)) we are able to integrate the angular velocity and calculate the change in the orientation and find the new quaternions:

$$\begin{pmatrix} \dfrac{d\varepsilon_1}{dt} \\ \dfrac{d\varepsilon_2}{dt} \\ \dfrac{d\varepsilon_3}{dt} \\ \dfrac{d\eta}{dt} \end{pmatrix} = \begin{pmatrix} \eta\omega_{x'} - \varepsilon_3\omega_{y'} + \varepsilon_2\omega_{z'} \\ \varepsilon_3\omega_{x'} + \eta\omega_{y'} - \varepsilon_1\omega_{z'} \\ -\varepsilon_2\omega_{x'} + \varepsilon_1\omega_{y'} + \eta\omega_{z'} \\ -\varepsilon_1\omega_{x'} - \varepsilon_2\omega_{y'} - \varepsilon_3\omega_{z'} \end{pmatrix} \tag{31}$$

Particles Characteristics

Table S1 contain a detailed description of the particles geometry and flow characteristics, including d_p - is the diameter of a sphere with the equivalent volume, a_p and b_p are the minor and major diameters of the fiber, d_{stk} is the fiber equivalent volume diameter given by Shapiro and Goldenberg (1993): $d_{stk} = 2a_p\sqrt{\frac{AR\ln\left(AR+\sqrt{AR^2-1}\right)}{\sqrt{AR^2-1}}}$, t_0 is the relaxation time calculated as $t_0 = \frac{\rho_p {d_{stk}}^2}{18\mu_g}$,

Where particle density $\rho_p = 1000\ kg/m^3$ and air dynamic viscosity $\mu_g = 1.26\,e-6\,kg/ms$, and Stk is the stokes number of fibers, calculated as $Stk = \frac{t_0 u_0}{D}$, where u_0 is the maximal velocity during peak inhalation and D is the average alveoli diameter. As the AR increase d_{stk} decrease, as well as the relaxation time and the Stk number of the fibers, meaning they are more convected, reach the flow velocity and follow stream lines.

Table S1. Particle non dimensional characteristics for all size and AR ensembles.

			**AR=**	**1.02**			
**d_p**	**a_p**	**b_p**	**d_Stk**	**Relexation time**	**Stk (acinus)**	**Stk (Bronchi)**	**Stk (TB)**
**1**	1	1	1.01	4.48E-05	4.07E-03	9.14E-02	2.99E-02
**2**	2	2	2.01	1.79E-04	1.63E-02	3.66E-01	1.19E-01
**3**	3	4	3.02	4.03E-04	3.67E-02	8.23E-01	2.69E-01
**4**	4	4	4.03	7.17E-04	6.52E-02	1.46E+00	4.78E-01
**5**	5	5	5.03	1.12E-03	1.02E-01	2.29E+00	7.47E-01
**6**	6	6	6.04	1.61E-03	1.47E-01	3.29E+00	1.08E+00
**7**	7	7	7.05	2.19E-03	2.00E-01	4.48E+00	1.46E+00
**10**	10	10	10.07	4.48E-03	4.07E-01	9.14E+00	2.99E+00
**20**	20	20	20.13	1.79E-02	1.63E+00	3.66E+01	1.19E+01
			**AR=**	3			
**d_p**	**a_p**	**b_p**	**d_Stk**	**Relexation time**	**Stk (acinus)**	**Stk (Bronchi)**	**Stk (TB)**
**1**	0.69	2.08	0.94	3.94E-05	3.58E-03	8.03E-02	2.62E-02
**2**	1.39	4.16	1.90	1.60E-04	1.45E-02	3.26E-01	1.06E-01
**3**	2.08	6.24	2.84	3.58E-04	3.25E-02	7.30E-01	2.38E-01
**4**	2.77	8.32	3.79	6.34E-04	5.77E-02	1.29E+00	4.23E-01
**5**	3.47	10.4	4.74	9.95E-04	9.05E-02	2.03E+00	6.64E-01
**6**	4.16	12.48	5.69	1.43E-03	1.30E-01	2.92E+00	9.54E-01
**7**	4.85	14.56	6.63	1.94E-03	1.77E-01	3.97E+00	1.30E+00
**10**	6.93	20.8	9.48	3.97E-03	3.61E-01	8.10E+00	2.65E+00
**20**	13.87	41.6	18.97	1.59E-02	1.45E+00	3.25E+01	1.06E+01
			**AR=**	10			
**d_p**	**a_p**	**b_p**	**d_Stk**	**Relexation time**	**Stk (acinus)**	**Stk (Bronchi)**	**Stk (TB)**
**1**	0.46	4.64	0.80	2.81E-05	2.56E-03	5.74E-02	1.88E-02
**2**	0.93	9.28	1.61	1.15E-04	1.05E-02	2.35E-01	7.67E-02
**3**	1.39	13.92	2.41	2.57E-04	2.34E-02	5.24E-01	1.71E-01
**4**	1.86	18.57	3.23	4.60E-04	4.18E-02	9.39E-01	3.07E-01
**5**	2.32	23.21	4.02	7.16E-04	6.51E-02	1.46E+00	4.77E-01
**6**	2.78	27.85	4.82	1.03E-03	9.34E-02	2.10E+00	6.85E-01
**7**	3.25	32.49	5.64	1.40E-03	1.28E-01	2.87E+00	9.37E-01
**10**	4.64	46.41	8.05	2.86E-03	2.60E-01	5.84E+00	1.91E+00
**20**	9.28	9.083	16.10	1.15E-02	1.04E+00	2.34E+01	7.64E+00
			**AR=**	30			
**d_p**	**a_p**	**b_p**	**d_Stk**	**Relexation time**	**Stk (acinus)**	**Stk (Bronchi)**	**Stk (TB)**
**1**	0.32	9.65	0.65	1.85E-05	1.69E-03	3.78E-02	1.24E-02
**2**	0.64	19.31	1.30	7.42E-05	6.74E-03	1.51E-01	4.95E-02
**3**	0.96	28.96	1.94	1.67E-04	1.52E-02	3.41E-01	1.11E-01
**4**	1.29	38.62	2.61	3.01E-04	2.74E-02	6.15E-01	2.01E-01
**5**	1.61	48.27	3.26	4.69E-04	4.27E-02	9.58E-01	3.13E-01
**6**	1.93	57.93	3.91	6.75E-04	6.13E-02	1.38E+00	4.50E-01
**7**	2.25	67.58	4.55	9.17E-04	8.33E-02	1.87E+00	6.11E-01
**10**	3.22	93.55	6.52	1.88E-03	1.71E-01	3.83E+00	1.25E+00

**20**	6.44	193.1	13.03	7.51E-03	6.83E-01	1.53E+01	5.01E+00

Table S2. Computational details and various properties of flow and particles used in the present simulations.

**Fluid Properties**	**Solver**	**Turbulence model**	**Inlet boundary conditions**	**Outlet boundary conditions**	**Mesh size**	**Time step**	**Pressure-velocity coupling**	**Momentum**
**Fluid Properties**	**Finite Volume Method (Software-Fluent)**	RANS based realizable *k*-ε	DPI inhalation flow rate	Fixed weighted flow rate	~2.2 M (Upper airway model) ~1.7 M (Bronchial tree model)	0.01 sec	SIMPLEC	Second order upwind
**Particle Properties**	**Solver**	**Particle Shape**	**Particle Size**	**Particle Density**	**Total particles released**	**Time step**	**Forces on particles**	**Injection time**
**Particle Properties**	Discrete element method (DEM)	Ellipsoid	1-20 μm	1000 kg/m^3	108000	2e-7 sec	Viscous drag, aerodynamic lift and gravity	0.45 – 0.6 sec

Converging the number of particles simulated in the upper airways model

We analysed the DE results of different number of randomized particles. According to the results presented in Figure S6, negligible difference was found between simulating 1,500 particles of the same group and simulating 3,000 particles of the same group.

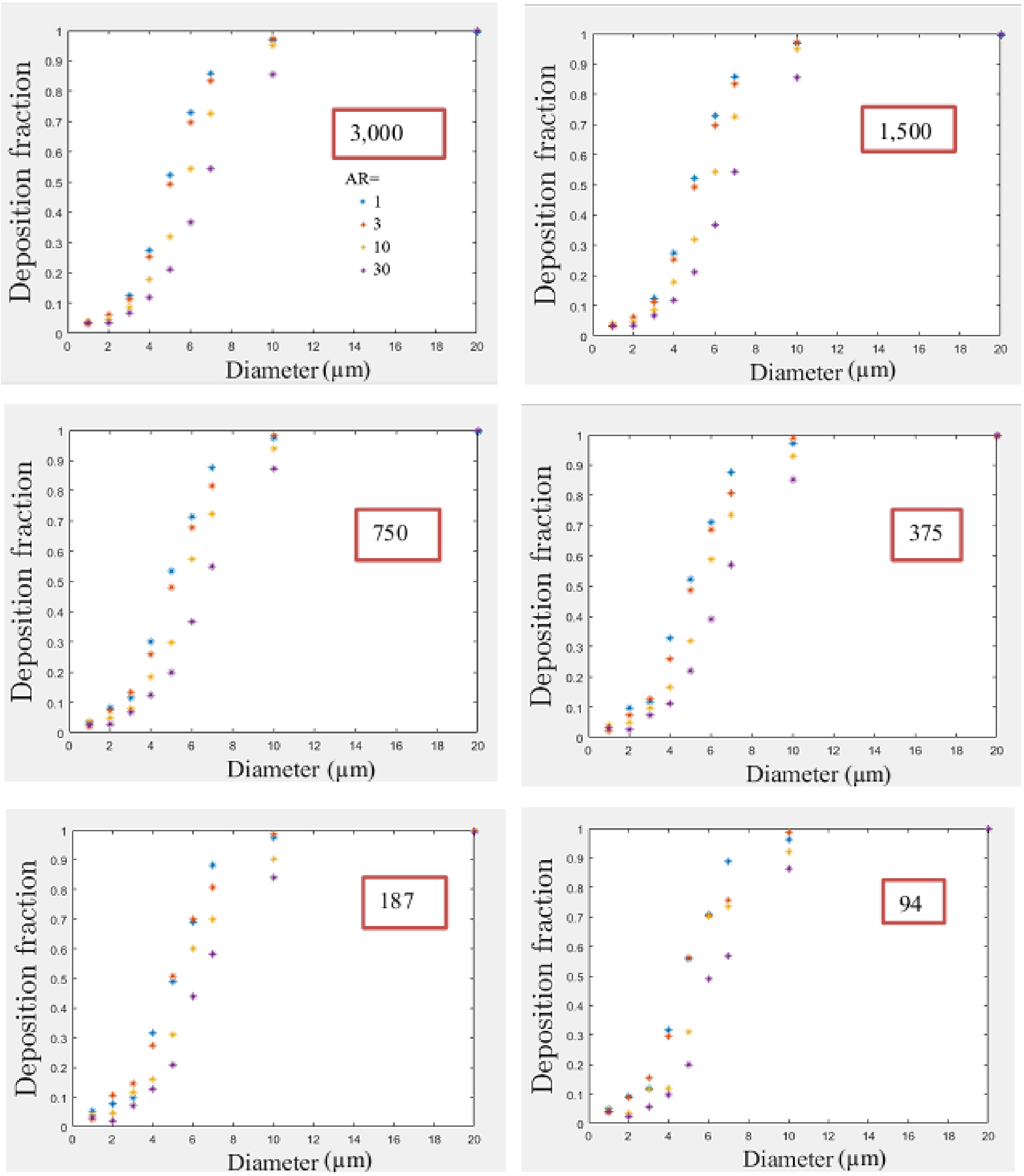

Figure S6. DE results of different number of particles, shown in each plot. Negligible difference was found between 1,500 and 3,000 particles results.

Validation for upper airways deposition efficiencies

Tian & Ahmadi, (2013)

We compared our upper airways deposition efficiencies to the work of Tian & Ahmadi [48] (see Figure S7).

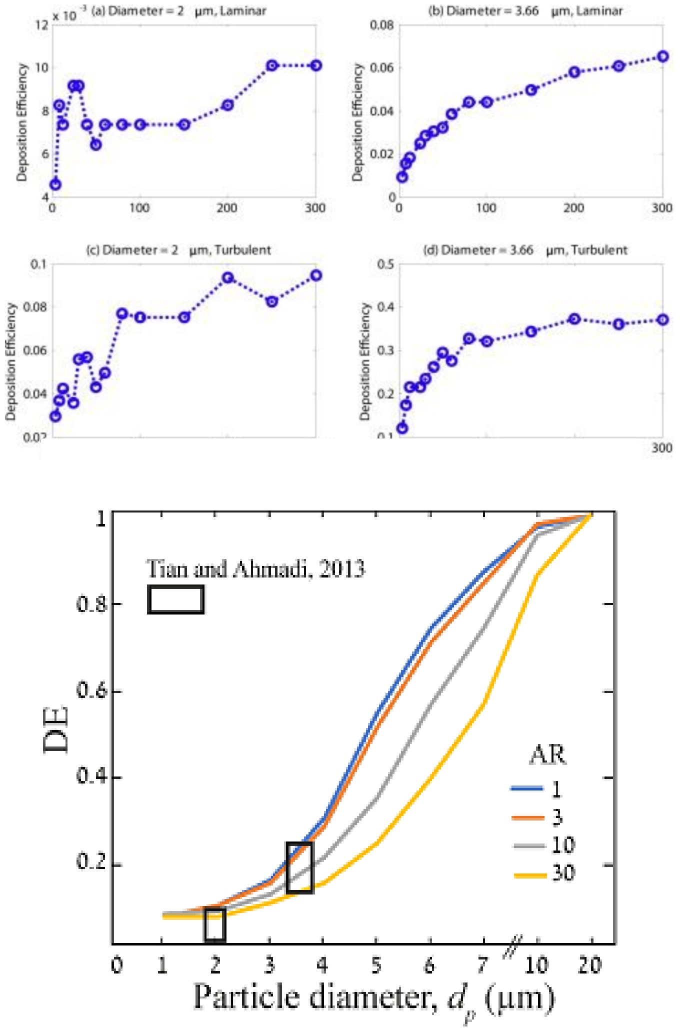

Figure S7. Validation of upper airways deposition against the work of Tian & Ahmadi [48] (shown on the left). On the right- the results of the current study match the black squares describing the results of Tian & Ahmadi [48].

Feng & Kleinstreuer(2013)

We compared our upper airways deposition efficiencies to the work of Feng & Kleinstreuer [18] and found a match between the current study results of fibers of AR=3 and AR=10 with $a_p = 1.83\ \mu m$. Our results did not match the results of AR=30 (see Figure S8).

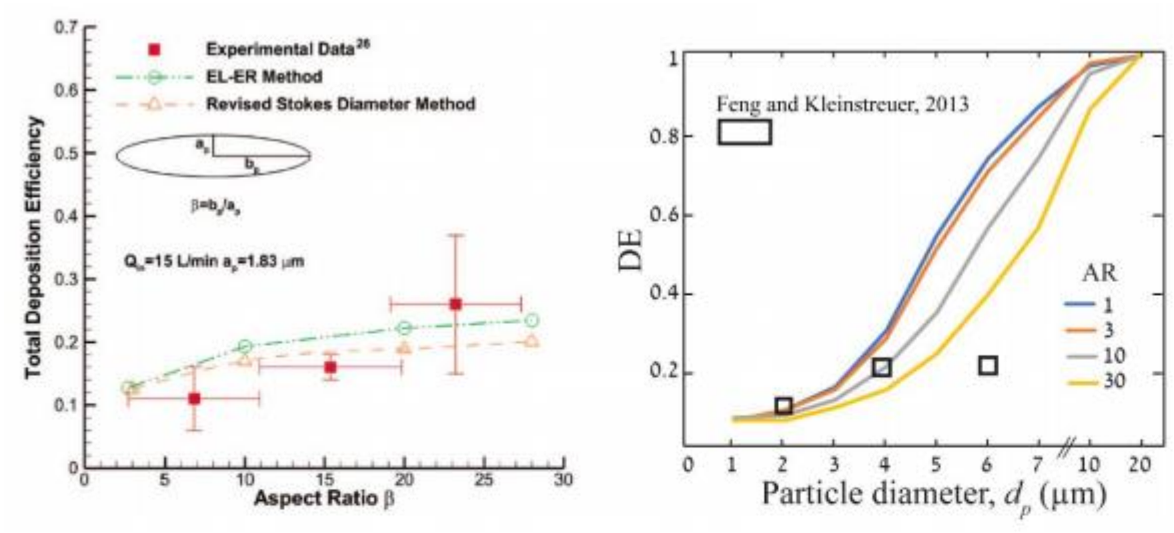

Figure S8. Validation of upper airways deposition against the work of Feng & Kleinstreuer [18] (left). In black squares, the matched results of AR=3,10 are shown in both plots.

Validation of the bronchi model

We compared our bronchial airways deposition efficiencies to the work of Koullapis *et al.* [23] and found a good match between the current study results of spheres to the range of particle sizes simulated (see Figure S9).

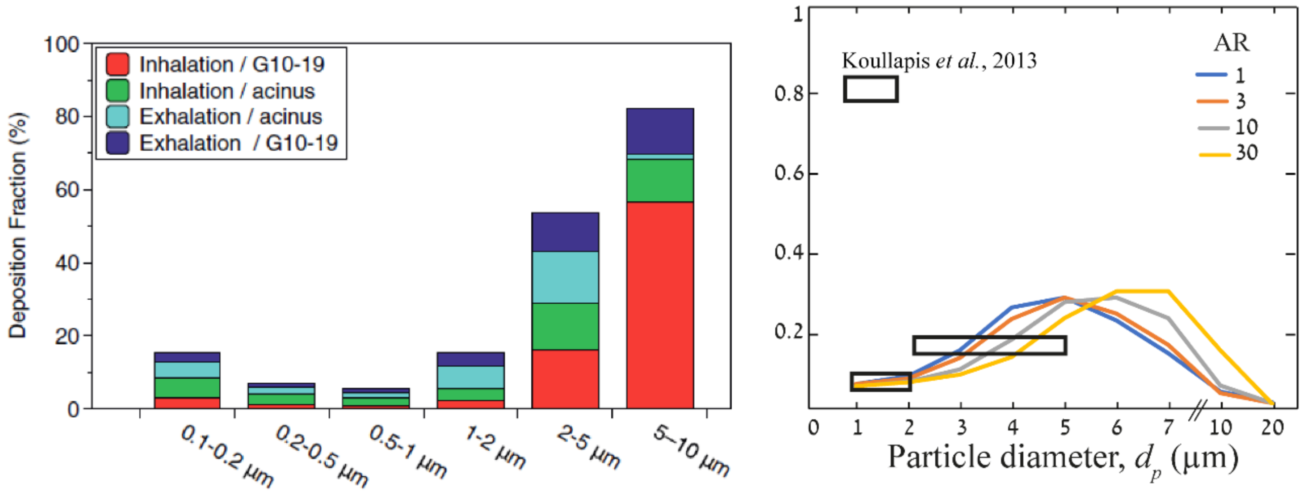

Figure S9. Validation of bronchial deposition against the work of Koullapis *et al.* [23] (left). In black squares, the matched results of spheres $\boldsymbol{d_p = 1 - 2\ \mu m, d_p = 2 - 5\ \mu m}$ are shown (right).
